# Effects of Hemodiafiltration and High Flux Hemodialysis on Nerve Excitability in End-Stage Kidney Disease

**DOI:** 10.1371/journal.pone.0059055

**Published:** 2013-03-11

**Authors:** Ria Arnold, Bruce A. Pussell, Timothy J. Pianta, Virginija Grinius, Cindy S-Y. Lin, Matthew C. Kiernan, James Howells, Meg J. Jardine, Arun V. Krishnan

**Affiliations:** 1 Translational Neuroscience Facility, University of New South Wales, Sydney, New South Wales, Australia; 2 Department of Nephrology Prince of Wales Hospital, Sydney, New South Wales, Australia; 3 Prince of Wales Clinical School, University of New South Wales, Sydney, New South Wales, Australia; 4 Neuroscience Research Australia, Sydney, New South Wales, Australia; 5 The University of Sydney and Institute of Clinical Neurosciences, Royal Prince Alfred Hospital, Sydney, New South Wales, Australia; 6 Department of Nephrology Concord Repatriation General Hospital and The George Institute for Global Health, Sydney, New South Wales, Australia; University of Toronto, Canada

## Abstract

**Objectives:**

Peripheral neuropathy is the most common neurological complication in end-stage kidney disease. While high flux hemodialysis (HFHD) and hemodiafiltration (HDF) have become the preferred options for extracorporeal dialysis therapy, the effects of these treatments on nerve excitability have not yet been examined.

**Methods:**

An observational proof-of-concept study of nerve excitability and neuropathy was undertaken in an incident dialysis population (n = 17) receiving either HFHD or HDF. Nerve excitability techniques were utilised to assess nerve ion channel function and membrane potential, in conjunction with clinical assessment and standard nerve conduction studies. A mathematical model of axonal excitability was used to investigate the underlying basis of the observed changes. Nerve excitability was recorded from the median nerve, before, during and after a single dialysis session and correlated with corresponding biochemical markers. Differences in nerve excitability were compared to normal controls with longitudinal follow-up over an 18 month period.

**Results:**

Nerve excitability was performed in patient cohorts treated with either HFHD (n = 9) or online HDF (n = 8), with similar neuropathy status. Nerve excitability measures in HDF-treated patients were significantly closer to normal values compared to HFHD patients obtained over the course of a dialysis session (*p<*0.05). Longitudinal studies revealed stability of nerve excitability findings, and thus maintenance of improved nerve function in the HDF group.

**Conclusions:**

This study has provided evidence that nerve excitability in HDF-treated patients is significantly closer to normal values prior to dialysis, across a single dialysis session and at longitudinal follow-up. These findings offer promise for the management of neuropathy in ESKD and should be confirmed in randomised trials.

## Introduction

Neurological complications are a major cause of physical disability in patients with end-stage kidney disease (ESKD). Of the many neurological complications that can occur in ESKD [1], peripheral neuropathy remains the most common long-term disorder [2]–[4]. Clinical features of neuropathy include sensory loss, paraesthesia, impaired vibration sense, reduced deep tendon reflexes, muscle wasting and weakness [4], [5]. Despite the considerable advances that have occurred in the use of renal replacement therapies (RRT), neuropathy rates remain high affecting 70 to 100% of dialysis patients [2]–[4], [6]. Moreover, neuropathy remains essentially untreatable in the majority of ESKD patients. As such, further investigation into the most beneficial treatment for ESKD patients with neuropathy is a matter of high priority.

The two preferred options for extracorporeal dialysis therapy among renal physicians are high flux hemodialysis (HFHD) and hemodiafiltration (HDF) [7]. While recent ongoing randomized trials have thus far failed to show survival benefit with HDF treatment, there remains widespread interest in the potential clinical advantages of HDF [8], [9]. HDF has been reported to achieve better clearance of “clinically relevant” middle molecules [10] and studies have suggested that on-line HDF may have a positive effect on inflammatory status [11], [12], oxidative stress [13] and small solute clearance [14]–[16]. Previous studies have not however addressed neurological function in patients treated with online HDF and HFHD forms of RRT.

While traditional nerve conduction studies demonstrate minimal change over a dialysis session [3], nerve excitability studies have demonstrated prominent abnormalities prior to dialysis that are rapidly reversed by dialysis [17]–[19]. These techniques are sophisticated neurophysiological measures that provide insights into ion channel function and membrane potential in human peripheral nerves. As such, nerve excitability techniques have been widely used in clinical neurophysiological research and have proven highly sensitive to the alterations in nerve function that occur with therapeutic interventions, such as hemodialysis [17], chemotherapy administration [20] and immunological therapy [21]. The present study was undertaken to investigate whether there were differences in peripheral nerve excitability studies in patients treated with HDF and HFHD.

## Methods

### Ethics Statement

The studies were approved by the South East Sydney Area Health Service Human Research Ethics Committee (Northern Section) and the Human Research Ethics Committee of the University of New South Wales. Participants gave written informed consent to the procedures in accordance with the Declaration of Helsinki.

An observational, proof-of-concept, clinical study of nerve excitability and neuropathy was undertaken in an incident dialysis population at a tertiary referral centre from 2010–2012. Eligibility criteria included ESKD patients aged 18–75 years, able to give informed consent and who were maintained on HFHD or HDF dialysis modality for a continuous period of at least 6 months. Sensory and motor nerve conduction studies, including sensory comparison studies, were undertaken in all patients to exclude the presence of carpal tunnel syndrome. Patients who had alterations in median sensory or motor conduction, suggestive of carpal tunnel syndrome, were excluded from the study. Study sample size was based on calculations of changes in threshold electrotonus parameters (α error level of 5% and a statistical power of 80%) from previous studies of nerve excitability undertaken in dialysis populations [17]–[19].

Clinical assessment and neurophysiological studies were undertaken in 17 ESKD patients receiving 4–6 hour, thrice-weekly RRT. Nine patients were treated with HFHD and eight with online post dilution HDF with at least 20 litres of fluid replacement. In order to minimise baseline differences, groups were matched for age, gender, disease duration, presence of diabetes and current neuropathy status. Neurophysiological tests were also undertaken in 20 age and gender matched normal subjects. The two study groups were similar in age, gender, neuropathy status, and diabetic status (Table 1). The causes of ESKD included diabetes, glomerulonephritis, lithium toxicity, amyloid, obstructive nephropathy, interstitial nephritis, reflux nephropathy and in four the cause was unknown. Longitudinal assessment of these tests was undertaken 18 months later in 13 of the 17 patients.

**Table 1:** Patient Characteristics.

	HDF	HFHD	*p*
**Demographics**			
Age	61 (14)	62 (14)	0.87
Sex	6M : 2F	7M : 2F	
Diabetes	4Y : 4N	4Y : 5N	
**Neuropathy Status**			
Total Neuropathy Score	9.9 (3.5)	7.3 (2.7)	0.46
Total Neuropathy Grade	1.7 (0.5)	1.1 (0.4)	0.46
Sural Nerve Amplitude (µV)	6.7 (2.0)	7.5 (2.3)	0.61
Tibial Motor Nerve Amplitude (mV)	4.3 (3.5)	5.8 (5.1)	0.66
**RRT measures**			
Years on RRT	3.9 (2.4)	4.2 (2.8)	0.79
Hours on RRT (per session)	4.9 (0.5)	4.7 (0.4)	0.54
Equilibrated KT/V	1.81 (0.44)	2.28 (0.76)	0.15
Urea Reduction Ratio	0.79 (0.06)	0.81 (0.13)	0.28
**Pre-RRT serum biochemistry**			
Sodium (mmol/L)	138(3)	139 (2)	0.70
Potassium (mmol/L)	4.5 (0.7)	5.3 (0.8)	0.03*
Chloride (mmol/L)	100 (3.7)	103 (3.8)	0.14
Bicarbonate (mmol/L)	25.6 (2.4)	23.6 (2.1)	0.08
Serum Urea Nitrogen (mg/dL)	61.1 (30.1)	65.8 (14.0)	0.68
Creatinine (µmol/L))	807 (344)	882 (236)	0.60
Calcium (mmol/L)	2.23 (0.17)	2.27 (0.15)	0.65
Magnesium (mmol/L)	1.01 (0.13)	1.09 (0.14)	0.28
Serum Phosphorus (mmol/L)	1.36 (0.61)	1.53 (0.50)	0.52
Parathyroid hormone (ng/L)	351 (380)	261 (305)	0.56

All values given as mean (±SD). *p<0.05 Biochemical values all taken pre-RRT after a 56 hour inter-dialytic period.

Enrolled patients had been maintained on their respective form of RRT consistently for the previous six months. The dialyzers used were Polyflux® 201H (surface area 2.1 m^2^) with either a Gambro® 200S dialysis machine for HFHD or a Gambro® AK 200Ultra for online HDF (Gambro, Hechingen, Germany). HDF machines dialysed against ultrapure water, while HFHD used pure water and both modalities used Gambro® Select Bag AX250G dialysis concentrate containing sodium (Na^+^) 140 mmol/L, bicarbonate 34 mmol/L, potassium (K^+^) 2.0 mmol/L, calcium 1.5 mmol/L, magnesium 0.50 mmol/L and glucose 1.0 g/L. All patients were adequately dialysed as verified by eKT/V (>1.05) and URR (>65%) [9], [22].

To ensure groups were matched for severity of pre-existing neuropathy, clinical neurological assessment was undertaken in all patients. Neuropathy was graded using a modified version of the Total Neuropathy Score [23]. This scale is a validated measure of neuropathy severity that assigns a neuropathy grade from 0 (no neuropathy) to 4 (disabling neuropathy), based on clinical features and the results of nerve conduction studies [23]. Nerve conduction studies were conducted using a Medelec Synergy system (Oxford Instruments, Abingdon, United Kingdom).

Motor nerve excitability studies were obtained from the non-fistula arm of ESKD patients before, during and after a single RRT session. All excitability recordings were conducted following a 56-hour interdialytic period. Compound muscle action potentials were recorded from the abductor pollicis brevis muscle following median nerve stimulation using previously published protocols [17], [19], [24]. Multiple excitability parameters were recorded, including threshold electrotonus and current threshold recordings which provide information on nodal and internodal conductances. The protocol also assessed recovery cycle parameters (refractoriness, superexcitability and late subexcitability), which provide information regarding changes in Na^+^ and K^+^ channel function and membrane potential [25]. Skin temperature was monitored close to the site of stimulation for the duration of the study. Serum electrolytes, urea, creatinine, calcium, magnesium, phosphate and parathyroid hormone were collected at time intervals corresponding to nerve excitability studies.

### Mathematical Modelling

An established model of axonal excitability [26]–[30] was used to assess whether changes in extracellular K^+^ concentration alone could account for the observed differences in excitability between the two groups of patients. This model consists of nodal and internodal compartments linked by a paranodal pathway through and under the myelin sheath [31]. Voltage-gated Na^+^ channels (both transient and persistent), K^+^ channels (slow and fast) were modelled on the nodal axolemma, while the internode incorporated both slow and fast K^+^ channels and the hyperpolarization-activated cation current *I*_h_. Separate leak conductances, Na^+^/K^+^-ATPase pump currents and axolemmal capacitances were modelled at both the node and internode.

### Statistical Analysis

All statistical analyses were performed using IBM SPSS Statistics Package version 20 (SPSS Inc, Chicago, Illinois) with statistical significance defined as *p≤*0.05. Clinical variables were expressed as mean±standard deviation (SD) and neurophysiological measures, figures and graphs are expressed as mean±standard error of the mean (SEM). Prior to analysis all nerve excitability data was checked for normality using Shapiro Wilks' test. Direct comparison of pre-RRT recordings with normal controls and analysis of between-group variables was undertaken using ANOVA or Kruskal-Wallis Test. To determine between group differences post-hoc analysis was undertaken. Two-factor mixed model ANOVA analysis was used to investigate differences throughout the dialysis session recordings. Correlation analyses were used to explore relationships between potential uremic toxins and nerve excitability parameters. Longitudinal analyses were undertaken using Wilcoxon Signed Rank test and Mann-Whitney U test was used for intention to treat analyses.

## Results

### Clinical Findings

Table 1 demonstrates the clinical and biochemical characteristics for HDF and HFHD patient groups. There were no significant differences between the two groups for age, gender, neuropathy status, and diabetic status. Additionally, there were no significant differences between groups for years on dialysis (HDF 3.9±2.4 years; HFHD 4.2±2.8 years), dialysis duration (HDF 4.9±0.5 hours; HFHD 4.7±0.4 hours) or dialysis adequacy as measured by URR (HDF 0.79±0.06; HFHD 0.81±0.13) or eKT/V (HDF 1.81±0.44; HFHD 2.28±0.76). In keeping with previous studies, the prevalence of neuropathy in this ESKD cohort (defined as a total neuropathy score >1) [23] was ∼70% [3], [4], [19]. The groups demonstrated similar severity of pre-existing neuropathy, with no significant baseline differences for total neuropathy score (HDF 9.9±3.5; HFHD 7.3±2.7) or total neuropathy grade (HDF 1.7±0.5; HFHD 1.1±0.4).

### Baseline Nerve Excitability Findings

**Figure 1:**
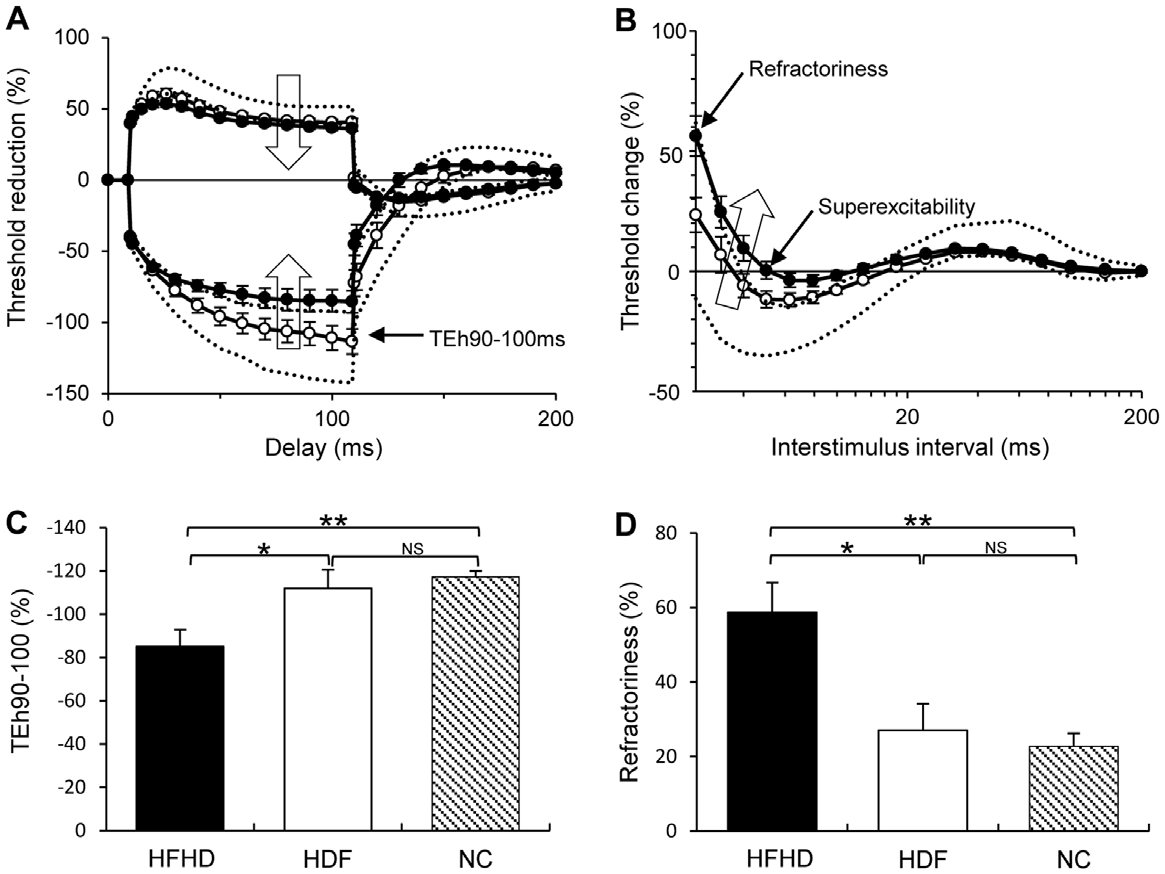
Pre-RRT nerve excitability recordings. Patients receiving hemodiafiltration (HDF) (empty circles) and standard high-flux HD (black filled circles) compared to 95% limits for healthy controls (dashed lines). (A) Depolarising and hyperpolarising threshold electrotonus curves. (B) Recovery cycle curve of excitability, large empty arrows indicate the direction of change with axonal depolarization. In both figures (A) and (B) the HDF group demonstrate results significantly closer to normal values than the HFHD group, specifically at hyperpolarising threshold electrotonus 90–100 ms (TEh90–100 ms), refractoriness and superexcitability. Figures (C) and (D) demonstrate the group differences in TEh90–100 ms and refractoriness in histogram format. *p<0.05, **p<0.001.

To assess acute changes in nerve excitability across a dialysis session, recordings were conducted before, during and after a single RRT session. When compared to normal controls (NC), pre-RRT recordings in both groups demonstrated multiple abnormalities with the overall pattern of change consistent with nerve membrane depolarization (Fig. 1) [17], [32], a finding that has been previously reported in studies of excitability in ESKD patients dialyzed with conventional semi-permeable membranes [17], [19]. Specifically, comparison across the three groups (NC, HDF and HFHD) demonstrated significant differences for multiple nerve parameters; refractoriness, a marker of Na^+^ channel inactivation [33], superexcitability, a marker of the function of fast K^+^ channels [33], depolarising threshold electrotonus at the 10–20 ms interval (TEd10–20), hyperpolarising threshold electrotonus at the 90–100 ms interval (TEh90–100 ms) and resting current threshold slope (all ANOVA *p* = 0.001), markers of internodal ion channel function and membrane potential [32] (Table 2). Taken together, reductions in these parameters are reflective of nerve membrane depolarization [32].

**Table 2:** Pre-RRT nerve excitability results for HDF and HFHD groups compared to controls.

	Normal controls		HDF		HFHD	ANOVA F, *p*
**Recovery Cycle**						
Refractoriness (%)		19.3(±2.9)	27.0(±7.2)	60.4(±8.4)a,b		15.4, <0.001
Superexcitability (%)		−21.7(±1.5)	−12.2(±2.3)^a^	−2.0(±2.5)a,b		29.0, <0.001
**Threshold Electrotonus**						
TEh (90–100 ms) (%)		−117(±3)	−112(±9)	−85(±8)a,b		9.7, <0.001
TEd (10–20 ms) (%)		68.4(±1.0)	59.6(±3.6)^a^	53.1(±2.4)^a^		17.7, <0.001
TEd (90–100 ms) (%)		45.4(±0.7)	40.6(±2.2)^a^	36.4(±1.6)^a^		12.2, <0.001
**Current Threshold**						
Resting I/V slope		0.60(±0.01)	0.67(±0.07)	0.84(±0.07)a,b		8.3, 0.001

All values given as mean (±SEM). By convention threshold electrotonus, refractoriness and superexcitability are expressed as percentage change in threshold. TEd, depolarising threshold electrotonus; TEh, hyperpolarising threshold electrotonus; I/V, current threshold.

a Significantly different to controls,

b significantly different to HDF.

Critically, further analysis revealed that pre-RRT values in the HDF group were not significantly different to controls for several neurophysiological measures sensitive to alterations in nerve membrane potential (Fig. 1), namely refractoriness, TEh90–100 ms and resting I/V slope (Table 2). In contrast, the HFHD group demonstrated significantly impaired nerve excitability when compared to both normal controls and the HDF group. This was noted for refractoriness (NC; HFHD, *p<*0.001: HFHD; HDF, *p<*0.05), TEh90–100 ms (NC; HFHD, *p<*0.001: HFHD; HDF, *p<*0.05) and resting IV slope (NC;HFHD, *p<*0.001: HFHD; HDF, *p<*0.05) (Fig. 1, Table 2). Superexcitability demonstrated significant differences across all three groups, with a progressive worsening from normal controls to HDF to HFHD (NC −21.7±1.5%; HDF −12.2±2.3%: HD −3.0±2.5%; *p<*0.05). While depolarising threshold electrotonus 10–20 and 90–100 ms values also demonstrated a progressive worsening from controls to HDF to HFHD, there was no significant difference between RRT modes (Table 2). Thus, in total, HDF-treated patients demonstrated improved nerve excitability following a 56 hour inter-dialytic period when compared to patients receiving HFHD.

In addition to the pre-RRT findings, the HDF group demonstrated significantly greater normalisation of nerve excitability when assessed across the duration of a dialysis session. While both groups demonstrated significant improvement of nerve excitability abnormalities during and post-RRT, these changes were greater in the HDF group (refractoriness, F = 4.87, *p<*0.05; superexcitability, F = 11.52, *p<*0.05; resting I/V slope, F = 5.39, *p<*0.05), compared to HFHD. This enhanced normalisation in the HDF group was further supported by significantly greater superexcitability in the HDF group when compared to the HFHD group in studies undertaken at the conclusion of RRT (HDF −23.1±2.0: HFHD −18.1±1.4; *p<*0.05).

### Correlation between Changes in Nerve Excitability and Levels of Uremic Toxins

**Figure 2:**
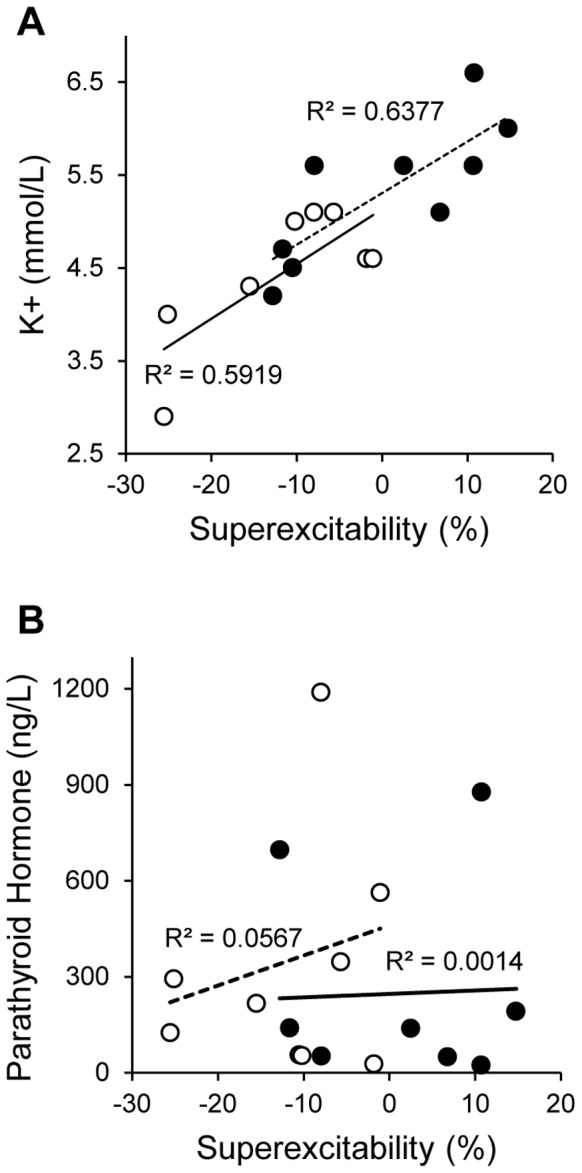
Relationship of pre-RRT bloods and superexcitability; a sensitive measure of membrane potential. (A) Pre-RRT serum K^+^ concentrations for both HDF (empty circles) and HFHD (black filled circles) demonstrated multiple significant correlations with nerve excitability parameters including superexcitability. (B) In contrast pre-RRT serum parathyroid hormone concentrations showed no correlation.

Correlations were undertaken to investigate the potential relationship between changes in nerve parameters and potential neurotoxins, including small solutes and middle molecules. There were no significant correlations noted between excitability parameters and concentrations of parathyroid hormone which has previously been suggested as a possible underlying cause of nerve dysfunction in ESKD [34], [35]. Pre-dialysis excitability parameters were however strongly correlated with pre-RRT serum K^+^ (Fig. 2), a finding that has been demonstrated in more recent studies of nerve excitability in dialysis [17]–[19]. Specifically, correlations were noted between pre-RRT serum K^+^ and refractoriness (HDF r = 0.76; *p<*0.05: HFHD r = 0.69; *p<*0.05) and superexcitability (HDF r = 0.77; *p<*0.05: HFHD r = 0.80; *p<*0.05). Serum K^+^ also strongly correlated with several threshold electrotonus parameters including TEh90–100 (HDF r = 0.59; *p = *0.12: HFHD r = 0.74; *p<*0.05). Importantly, pre-RRT serum K^+^ concentration was significantly different between the groups (HDF 4.5±0.7 mmol/L: HFHD 5.3±0.8 mmol/L; *p<*0.05), a finding that was also noted on longitudinal follow-up (HDF 4.7±0.2 mmol/L: HFHD 5.6+0.6 mmol/L; *p<*0.05). Aside from K^+^, there were no significant differences between the groups for other biochemical measures (Table 1), including urea (HDF 21.8+3.8 mmol/L; HFHD 23.5+1.7 mmol/L), creatinine (HDF 807.4±121.7 µmol/L; HFHD 882.4±78.7 µmol/L) and parathyroid hormone (HDF 351±380 ng/L; HFHD 261±305 ng/L).

### Longitudinal Assessment of Nerve Excitability

**Figure 3:**
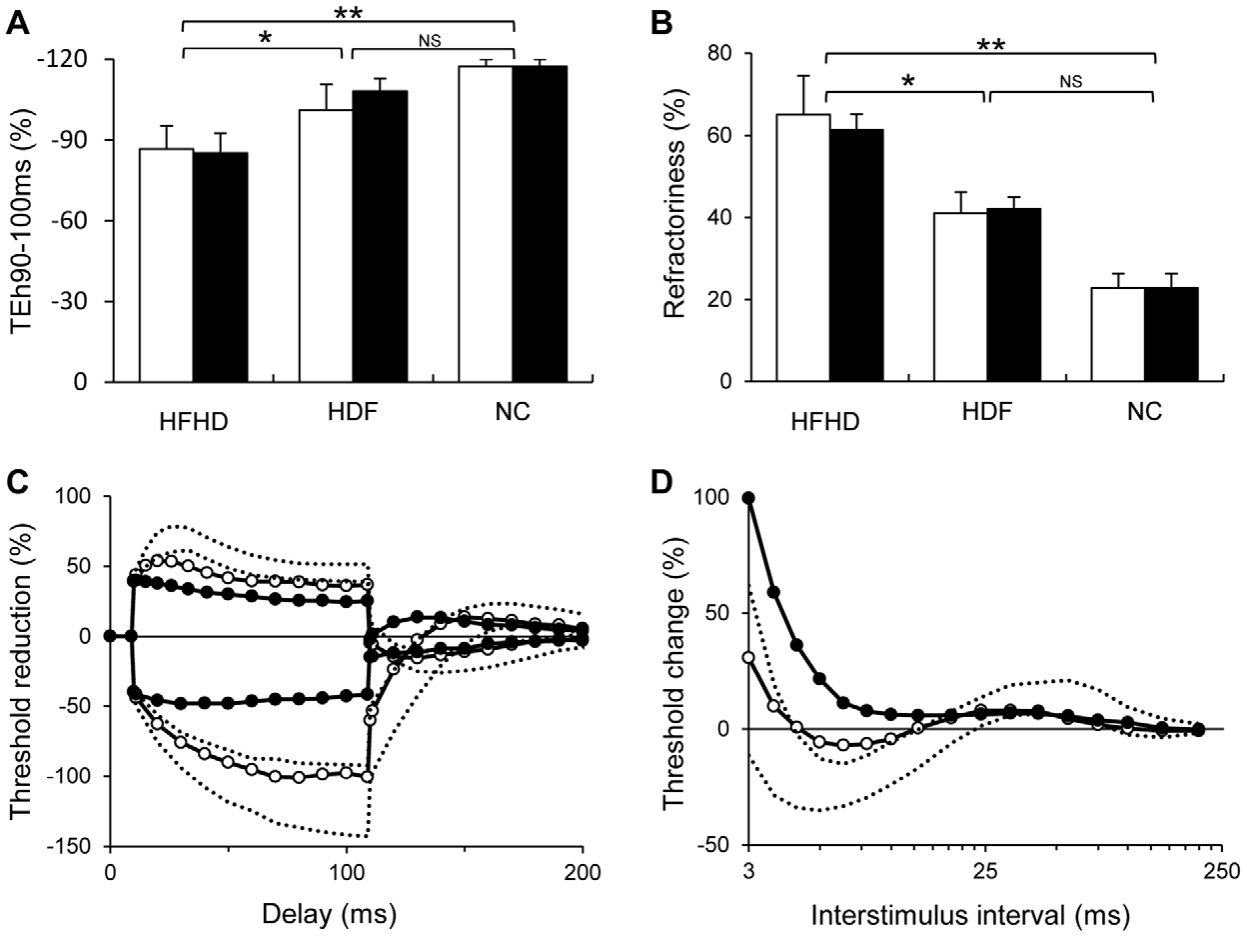
Longitudinal follow-up 18 months of patients who remained on their respective treatment (HDF n = 4 and HFHD n = 7). Panels (A) and (B) demonstrate a relative stability of nerve excitability results in patients who remained on their respective treatments at baseline (white bars) and longitudinal follow-up (black bars). Additionally these graphs depict the sustained significant difference between HDF and HFHD in both hyperpolarising threshold electrotonus 90–100 ms (TEh90–100 ms) and refractoriness. *p<0.05, **p<0.001. Panels (C) and (D) show the results of a single patient switched from HDF to HFHD. Threshold electrotonus (C) and recovery cycle parameters (D) demonstrate the profound abnormalities at longitudinal follow-up on HFHD.

Longitudinal assessments were conducted at 18 months on 13 patients. Of the four remaining patients, one received a renal transplant, two died and one declined further testing. There were no significant differences in baseline values between patients lost to follow-up those in whom longitudinal follow-up was undertaken, including serum K^+^ (4.9 mmol/L: 5.1 mmol/L), TNS (5.7∶9.4) and dialysis hours (4.5 hours: 4.8 hours) respectively. Of the original HDF group, four had remained on HDF while two were switched to HFHD, due to changes in equipment availability. Excitability values in both HDF and HFHD groups demonstrated no significant difference from those obtained at baseline (Fig. 3). Importantly, HDF patients maintained significant improvements in nerve excitability, when compared to the HFHD group, with pre-RRT recordings demonstrating sustained differences in superexcitability (*p<*0.05) and TEh 90–100 ms (*p<*0.05). When these variables were assessed using intention to treat analysis, significant differences remained between the two groups (superexcitability, *p = *0.05; TEh90–100, *p = *0.05). In two patients who were changed from HDF to HFHD, deterioration in nerve excitability was noted at 18-month follow-up, with no change in lower limb motor and sensory nerve conduction amplitudes or latency (Patient #1 Sural 22.4 µV, 57 m/s vs 24.0 µV, 56 m/s and distal tibial motor amplitude 10.8 mV, 4.6 m/s vs 11.6 mV, 4.5 m/s; Patient #2 had no measurable distal tibial motor or sural response at both time points). In patient #1 (a non-diabetic), the deterioration in neurophysiological parameters was accompanied by the onset of diffuse sensory neuropathic symptoms.

### Mathematical Modelling

**Figure 4:**
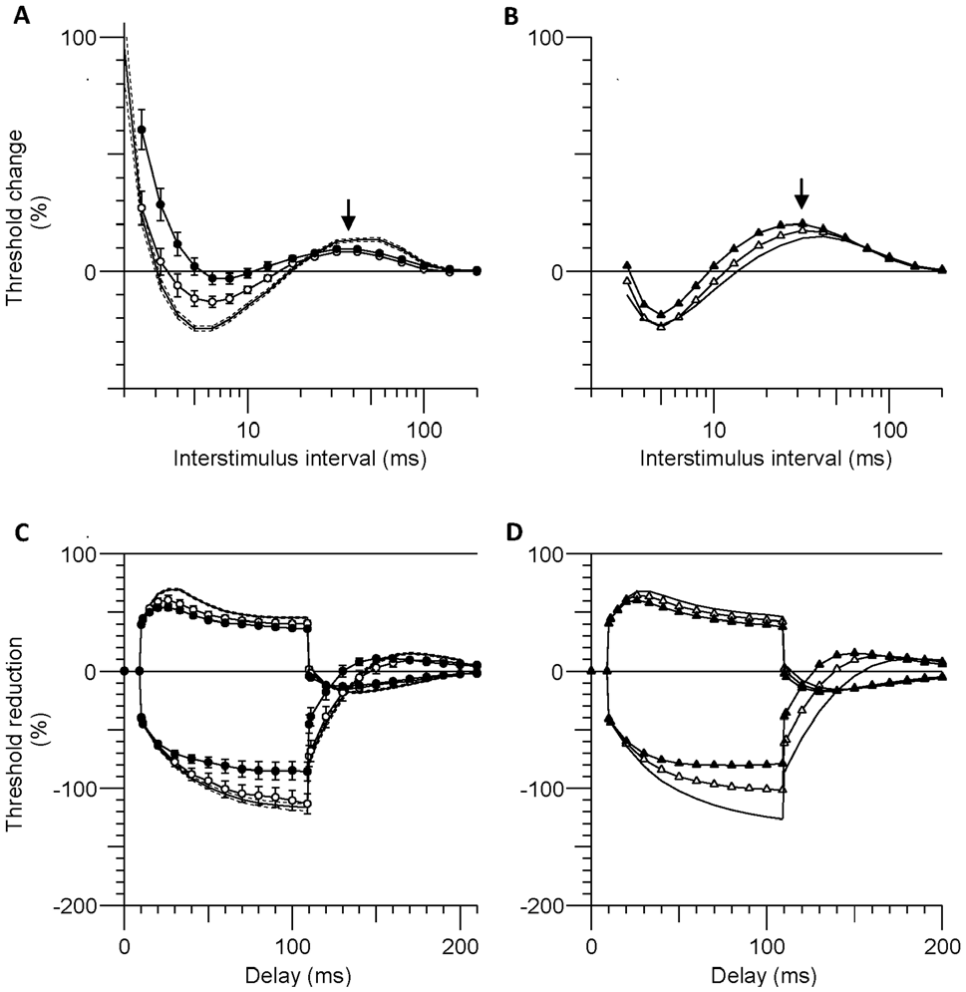
Mathematical model of depolarized axons. The observed excitability (mean ± SEM) is shown in panels (A) and (C) comparing HFHD patients (filled circles) and HDF (empty circles) to the healthy controls (solid and dashed lines). Panels (B) and (D) compare the modelled excitability of a healthy human motor axon (solid line) to the same model with: [K^+^]_o_ increased to 5.3 mmol/L (empty triangles); and with [K^+^]_o_  = 5.3 mmol/L and resting membrane potential further depolarized by 1 mV (filled triangles). The model provides a good fit to the changes in the threshold electrotonus data (C,D) but not the recovery cycle (A,B), particularly the extent of the changes in the refractory period, superexcitability and late subexcitability. Late subexcitability increased in the model (arrow in B) and decreased in the ESKD patients (arrow in A).

A model of human motor axons was used to explore the underlying mechanisms for the differences between the two patient groups and the healthy controls. In the model, an increase in extracellular K^+^ from 4.5 to 5.3 mmol/L alone depolarized resting membrane potential (RMP) by 2.8 mV, with the modelled changes in both threshold electrotonus and the recovery cycle consistent with depolarization (empty triangles in Fig. 4B & D). An additional 1-mV depolarization of RMP modelled threshold electrotonus better for the HFHD data (filled triangles in Fig. 4D). Pure depolarization of RMP however increased late subexcitability, in agreement with the findings of Kiernan and Bostock [2000] [32]. However in the ESKD patients subexcitability was decreased, and there were smaller changes in the other recovery cycle measures (compare arrows in Fig. 4B & A; see discussion).

## Discussion

The present study investigated the effects of HDF and HFHD on peripheral nerve excitability in incident ESKD patients. The study has demonstrated that neurophysiological measures of nerve function, specifically those sensitive to changes in nerve membrane potential, were significantly closer to normal in patients receiving HDF compared to the HFHD group. Furthermore, patients who remained on HDF maintained a more normal study profile compared to those on HFHD at long-term follow-up.

Overall, the pattern of change in excitability measures was consistent with nerve membrane depolarisation [32], an abnormality that has been demonstrated in previous studies of ESKD patients, undertaken during the era of conventional semi-permeable membranes [17]–[19]. Greater normality of nerve excitability results was noted in pre-RRT recordings and following a single RRT session in the HDF group, suggesting that HDF may achieve greater clearance of neurotoxic substances.

The implications of these findings are significant as maintenance of nerve membrane potential is essential for normal biochemical homeostasis and therefore axonal survival. [36], [37]. Chronic changes in neural excitability over time may trigger a cascade of events leading to axonal degeneration and development of symptoms of clinical neuropathy, such as numbness and muscle weakness, major causes of disability in ESKD [38]. Moreover, previous studies have demonstrated a direct relationship between neuropathic symptoms and pre-RRT excitability values [18]. The limitations of this study, including the small samples size and the largely cross-sectional nature of the data, suggest that longitudinal studies investigating the effects of nerve excitability changes and dialysis mode on the development of neuropathy are required.

While the HDF group demonstrated greater normality of nerve excitability, the underlying basis for this remains unclear. A potential explanation for the differential effects of HDF and HFHD on nerve excitability relates to the correlation that was noted between parameters of nerve excitability and serum K^+^ concentration. This correlation is consistent with the findings of recent studies that have suggested that hyperkalaemia may play an important role in the development of neuropathy in ESKD [17]–[19], [39]. Despite these lines of evidence, a causal relationship between elevated serum K^+^ concentration and neurological dysfunction has not yet been established. Moreover, mathematical modelling of data in the present study suggested that while changes in serum K^+^ may have played a role in the development of the abnormalities, the changes in excitability in the HFHD group were not explicable solely by increased K^+^ concentration. In particular, the significantly reduced super- and late sub- excitability in the ESKD patients may be due to a smaller time-integral of the action current, possibly a consequence of smaller Na^+^ currents [27], [30].

Given that both HDF and HFHD treatments in this study used the same membrane (Polyflux® 201H ), dialysate flow rate (Q_D_ 500 mL/min) and dialysate concentrations of K^+^, Na^+^, bicarbonate, calcium, magnesium and glucose, another potential explanation for the differences may relate to the dilutional effects of hemofiltration. The ultrafiltration setting in HDF allows for greater hemofiltration to occur in addition to the hemodialysis component. HDF is achieved with inline fluid replacement and ultrafiltration (UF) of 60 mL/min compared to UF of 0 mL/min for HFHD. Backfiltration of up to 10 litres can be achieved with HFHD whereas the hemofiltration in HDF delivers an average of 20 litres of inline fluid re-infused, which possibly contributes to a dilutional effect on overall clearances.

While previous studies have emphasised the role of middle molecules as a cause of nerve dysfunction in ESKD [40], [41], there was no significant correlation between PTH, a middle molecule assayed in this study, and pre-RRT measures of nerve excitability. However, while K^+^ was closely related to pre-RRT excitability differences, it cannot explain the during and post-RRT differences between groups. Furthermore, mathematical modelling demonstrated that an additional 1 mV depolarization was required to better model the threshold electrotonus data obtained in HFHD patients, suggesting that a synergistic effect of another neurotoxin may be required to induce nerve dysfunction in ESKD, a view that has been supported by previous neurophysiological studies [19], [42]. Given the known superiority of HDF in terms of middle molecule clearance and inflammatory status [12]–[15], future studies may need to explore a wider range of middle molecules and inflammatory mediators as potential contributors to nerve dysfunction in ESKD patients.

In conclusion, the present study provides evidence that ESKD patients treated with HDF demonstrate more normal nerve excitability both prior to RRT, across a single RRT session and at long-term follow-up compared to HFHD. These results suggest that HDF may help maintain normal nerve excitability in ESKD. The use of HDF for the clinical management of neuropathy in ESKD should be investigated further in large scale randomised trials.
